# *In vitro* models as tools for screening treatment options of head and neck cancer

**DOI:** 10.3389/fmed.2022.971726

**Published:** 2022-09-07

**Authors:** Barbara Seliger, Ahmed Al-Samadi, Bo Yang, Tuula Salo, Claudia Wickenhauser

**Affiliations:** ^1^Institute of Medical Immunology, Martin Luther University Halle-Wittenberg, Halle, Germany; ^2^Fraunhofer Institute for Cell Therapy and Immunology, Leipzig, Germany; ^3^Department of Oral and Maxillofacial Diseases, Clinicum, Faculty of Medicine, University of Helsinki, Helsinki, Finland; ^4^Translational Immunology Research Program, Research Program Unit, University of Helsinki, Helsinki, Finland; ^5^Cancer Research and Translational Medicine Research Unit, University of Oulu, Oulu, Finland; ^6^Medical Research Center Oulu, Oulu University Hospital, University of Oulu, Oulu, Finland; ^7^Institute of Pathology, Martin Luther University Halle-Wittenberg, Halle, Germany

**Keywords:** HNSCC, therapy, *in vitro* model, 3D culture, organoid, microfluidic chips

## Abstract

Various *in vitro* models using primary and established 2- and 3-dimensional cultures, multicellular tumor spheroids, standardized tumor slice cultures, tumor organoids, and microfluidic systems obtained from tumor lesions/biopsies of head and neck cancer (HNC) have been employed for exploring and monitoring treatment options. All of these *in vitro* models are to a different degree able to capture the diversity of tumors, recapitulate the disease genetically, histologically, and functionally and retain their tumorigenic potential upon xenotransplantation. The models were used for the characterization of the malignant features of the tumors and for *in vitro* screens of drugs approved for the treatment of HNC, including chemotherapy and radiotherapy as well as recently developed targeted therapies and immunotherapies, or for novel treatments not yet licensed for these tumor entities. The implementation of the best suitable model will enlarge our knowledge of the oncogenic properties of HNC, expand the drug repertoire and help to develop individually tailored treatment strategies resulting in the translation of these findings into the clinic. This review summarizes the different approaches using preclinical *in vitro* systems with their advantages and disadvantages and their implementation as preclinical platforms to predict disease course, evaluate biomarkers and test therapy efficacy.

## Introduction

Head and neck cancer (HNC) comprises neoplasms of the oral cavity, pharynx, and larynx and is the 8th leading cause of cancer worldwide with an incidence of >600.000 new cases and 300.000 deaths per year (1, 2). Well-known risk factors of HNCs are alcohol and tobacco consumption as well as human papilloma virus (HPV) infection, predominantly affecting the oropharyngeal region (3–6). HPV^+^ head and neck squamous cell carcinoma (HNSCC) is a genetically distinct carcinoma subgroup with a better prognosis than HPV^–^ HNSCC. Treatment of HNSCC is difficult due to their anatomic location that complicates surgery, its intratumoral heterogeneity and its highly variable treatment response.

The standard of care treatment of primary HNSCC in a locally advanced setting includes the surgical resection of the primary tumor and the regional lymph nodes in combination with adjuvant radiotherapy in the presence or absence of platinum-based chemotherapy. As an alternative, concurrent chemo-/radiotherapy is administered. Despite these treatment options are effective, they cannot prevent high rates of local recurrent tumors (7). Therefore, genetic screening by next-generation sequencing was used to identify patient-specific mutations as a rational for the development of a personalized therapy. As the epidermal growth factor receptor (EGF-R) was found to be frequently mutated and/or overexpressed in HNSCC, which was associated with a rather adverse prognosis (8), chemotherapy in combination with the EGF-R blocking antibody cetuximab is currently employed for the treatment of recurrent disease, but with moderate success rates.

Recently, the most promising treatment options for HNSCC used two antibodies directed against the immune checkpoint (ICP) molecule programed death receptor 1 (PD-1), nivolumab and pembrolizumab (9–12). These immune oncological treatments represent landmark in HNSCC therapy leading to a prolonged overall survival (OS) and therefore have been included in the guidelines for the treatment of recurrent or metastatic HNSCC. However, the problem of high recurrence rates persists and the success of immunotherapy combined with standard chemotherapy and radiation is still limited, which might be influenced by the pre-existing TME and its alterations during therapy (13, 14). These data underscore the requirement for new and effective HNSCC stratification strategies to optimize individual therapies and for the development of *ex vivo* strategies to monitor treatment responses. In the context of promising immunotherapy approaches as new treatment options for HNSCC, the lack of proper models/test platforms linking the specific or individual tumor biology and immunology research with clinical diagnosis, prognosis, and treatment strategies severely hampers the allocation of novel therapeutic approaches and drugs (15). Despite a long tradition of tumor tissue cultures, the crucial aspect of any preclinical model system to mimic human cancer development remains a challenge. Thus, there is an urgent need of models that reproduce the genomic heterogeneity of human cancer and develop a milieu that incorporates the multitude of immune and stromal cell populations and soluble factors that form the complex tumor microenvironment (TME).

## The problem of treatment resistance

Meta-analyses data strongly support the paradigm shift toward immunotherapy in relapsed HNSCC patients and clearly show that the immune checkpoint inhibitors (ICPi) recognizing PD-1 for programed death ligand 1 (PD-L1) improve OS in the second-line setting (16). Unfortunately, not all immunotherapy agents deliver equal survival outcomes in similar clinical settings and there is a serious risk for immune-associated toxicities and hyper progression during therapy (17, 18). Especially the last-mentioned risk of hyper progression indicates that inhibitor treatment is due to a combination of innate, adaptive or quickly acquired *de novo* resistances (19, 20). This frustrating observation suggests that immunotherapy is not a “one-size-fits all” approach. To estimate the biological behavior of the individual tumors next to the immunohistochemical evaluation of the presence of PD-L1, the composition of the immune cell infiltration, the mutational burden and/or the expression profile of immune-associated genes are currently being investigated in HNSCC (21–23). However, the determination of the complex landscape associated with genomic alterations includes far more parameters all important for prediction of HNSCC patients’ prognosis (24), and so far, no valid predictive markers for full monitoring of (immuno)therapy response in HNSCC have been established. Consistent with this statement, despite PD-L1 expression has been proposed as a prognostic biomarker in multiple trials (25), not all meta-analyses found a valid association between PD-L1 expression and treatment response (26, 27). This variable response and resistance to iCPI might be due to its unstable expression, the inaccuracy to detect PD-L1 expression on the tumor cell surface in 2D slides as well as the use of only baseline archival tumor material (28).

Taken together, the expectations of a suitable biomarker testing assume an increased knowledge about the immune resistance mechanisms allowing the prediction, which mode of immunotherapy can contribute to HNSCC patients’ survival. The identification of such suitable biomarkers is important to both improve the rational of treatment strategies and to avoid atypical response patterns with hyper progression in immune oncological therapies.

## *In vitro* models for monitoring treatment efficacy

Since establishing the cell culture technology in cancer research, scientists have developed several *in vitro* models to study tumor features including growth properties, immunogenicity as well as response to various treatment options. In this context, simulation of the TME is one of the primary goals. While this goal remains challenging, significant advancements were achieved, particularly after realizing the importance of the extracellular matrix and tumor stroma cells in shaping cancer cell behavior in several ways, including proliferation, migration, invasion, metastasis formation, and responses to anti-cancer treatments. Fundamentally, the inadequacy of using a simplified model of a two-dimensional (2D) plastic cell culture to study cancer is becoming more widely accepted and the results from using these 2D cultures might be inaccurate. Therefore, several more advanced models were developed to mimic essential features of the tumors including their differentiation properties, their microarchitecture, cellular variety and the TME (29). These elaborated models include spheroids (30), the sandwich assay (31), tumor slice cultures (32), organoids (33), and microfluidic systems (34). They all allow better than 2D systems to study the complexity of the tumors, their TME and their susceptibility to therapeutic intervention. While these assays significantly differ from each other and each harbors advantages and limitations, they all share the ability to provide an extracellular matrix (ECM) to cancer cells and to hold the possibility of co-culturing cancer and stroma cells. It is noteworthy that also mathematical modeling was used to predict individualized therapies (35, 36).

**FIGURE 1:**
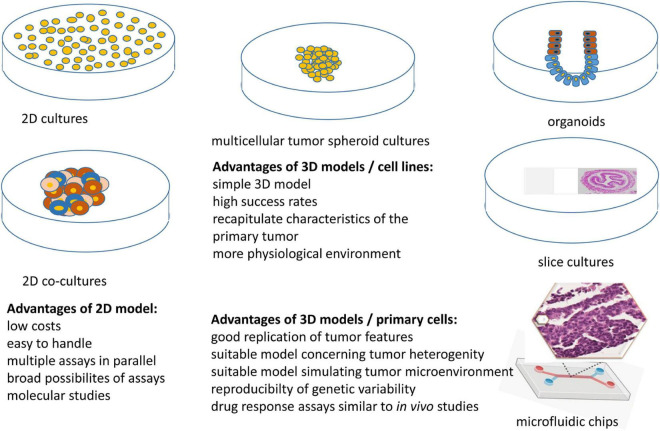
Distinct *in vitro* models for monitoring treatment efficacy.

Due to the development of new therapy regimens, such as targeted therapies and immunotherapies alone or in combination with standard therapies, the implementation of appropriate cell culture models rekindled the attention, those that grow in three-dimensional (3D) architecture and comprise the TME. Using these models for testing different treatment strategies, the outcome of the individual patient can be analyzed prior to and/or during treatment and will help to assign patients to their optimal therapy and identify intrinsic and extrinsic resistances. In the following paragraphs, next to a brief statement on the use of mouse models, the different *in vitro* systems, their establishment and use in HNSCC will be described (Figure 1 and Table 1).

**TABLE 1:** Different *in vitro* model systems as tool for monitoring growth properties, tumor immune cell interaction, and treatment options.

Parameters	2D models	3D models/cell lines	3D models/primary cells	Microfluidic chip
Costs	Low	Intermediate	Rather high	Rather high
Application	Easy to handle	More difficult to handle	Challenging	Challenging
Examination time	Multiple assays in parallel	More time consuming	Time consuming	Screening time consuming
Success rates	Low with primary cells	High	Moderate	Moderate
Similarity to the complexity of primary tumors	Rather low	Moderate	High	High
Suitability for drug response assays	Rather low	Moderate	High	High

## *In vivo* models—Advantages, disadvantages, and ethical aspects

Next to *in vitro* models for cancer research, HNSCC xenograft mouse models, which mimic the physiological and pathophysiological complexity of tumors, were already introduced more than 30 years ago (37–39). For many years these models represent the industrial gold standard for the development of cytotoxic cancer therapies and targeted therapies and for monitoring their efficacy employing human cancer cell lines/tumor tissues engrafted into immunocompromised mice to evaluate pharmacology, efficacy, and safety profiles of the agents to be tested (40–42).

Furthermore, transgenic mouse models have been developed to understand molecular drivers of HNSCC tumorigenesis (43, 44). To study the efficacy of targeted therapies, and in particular immunotherapies, unique issues for the development of *in vivo* models must be considered, which include model systems with a functionally intact immune system. The inherent heterogeneity and adaptability of the immune system might explain the relative success or failure of immunotherapies, since it is constantly adapting and evolving along with the tumor. Therefore, reliable preclinical mouse models recapitulating this adaptability in the presence of tumor heterogeneity remain a major impediment in the development of cancer immunotherapies and are urgently required for monitoring the efficacy of immunotherapies and understanding the resistance mechanisms (45, 46). So far, only a limited number of proper animal models for immunological research have been developed, while *in vitro* experiments have their limitations regarding the analysis of drug resistance and relapse (47). Recently, the tumorigenic HNSCC cell line “JC1” and the tumorigenicity-enhanced cell line “JC1-2” were generated as a model for monitoring the *in vivo* capacity of tumor development and drug sensitivity. While transplanted tumors derived from JC1 cells could only grow in immune deficient nude mice, tumors derived from JC1-2 cells could grow in immune competent C57BL/6 mice. Both cell lines had a microsatellite stable (MSS) phenotype and were responsive to interferon (IFN)-γ. Comparing orthotopic and heterotopic mouse tumor models of JC1-2 cells, an increased immune response in the microenvironment of the orthotopic model was found suggesting that this syngeneic model might be suitable for a better delineation of interactions between HNSCC and lymphocytes and the exploration of potential (immuno)therapeutic targets for HNSCC.

In addition, the implementation of *in vitro* models is also much more ethical for drug monitoring compared to animal models. Mouse models have been utilized for several years in cancer research and have served as the primary players in progress in such research. However, evidence is accumulating that the use of animal models to simulate human cancer carries major drawbacks due to tissue composition differences between species. In addition, studying the immunological aspects of cancer progression in mice has proved challenging (48). Drawbacks of animal studies include high costs, the time required for the experiments and highly educated personal. These drawbacks of mouse models are supported by the failure of most anti-cancer compounds, which successfully passed preclinical animal testing (49). Therefore, there is a clear need to find a suitable replacement for animal models, particularly given current wide support of the 3Rs principles–that is replacement, reduction and refinement of human animal research (50). Since the implementation of *in vitro* models are more ethical for drug monitoring compared to animal models, they emerged as promising candidates to replace or at least reduce reliance on animal models.

## Two-dimensional cultures of head and neck squamous cell carcinoma

Traditionally, cell culture experiments were performed with cells grown in a two-dimensional (2D) fashion on tissue culture plastic as adherent monolayers. Although this setting represents an accepted procedure with standardized protocols, low maintenance costs and well-preserved access of cells to nutrients and oxygen, it is well-known that 2D cultures have many limitations, such as a different cell morphology, no natural structure of the tumor, the lack of a nutrient and oxygen gradient physiologically present in the tumor tissues and no tumor specific complex cell-cell or cell-matrix interactions (51, 52). Furthermore, the success rates to establish HNSCC-derived cell lines from primary tumor cells range from 11 to 33% (53). Nevertheless, *in vitro* drug screens of 2D cell lines have been used to characterize the variability in drug response among tumors and as a tool to understand therapy resistance mechanisms of tumor cells (54, 55), but the value for testing the *in vitro* treatment efficacy is limited. One important above-mentioned limitation of the 2D assays is that the tumor cells are unphysiologically exposed in an equal manner to oxygen, nutrients, and drugs, which is not the case in 3D cultures (spheroids, organotypic cultures, and organoids) and in the *in vivo* setting. In addition, in 2D monocultures, the impact of cell-cell interactions is missing and thus the informative value of the *in vitro* treatment efficacy is limited. These drawbacks are also reflected by differences of 2D and 3D cultures in response to radiation, EGFR inhibition, or cisplatin treatment (56, 57).

## Three-dimensional cultures/multicellular tumor spheroids of head and neck squamous cell carcinoma

To overcome limitations of the 2D systems, HNSCC cell lines and tumor lesions have been replated in a three-dimensional (3D) format. Multicellular tumor spheroids (MCTS) (58), already established for HNSCC some decades ago, are the simplest 3D model. They represent cell aggregates grown from single cell cultures or tissue fragments and consist of an outer layer of proliferating cells and an inside layer of mainly quiescent cells with necrotic areas in the center (59). The success rate of MCTS ranges between 50 and 100% of HNSCC originated at different locations with a range of culture of time between 4 and 21 days (60). Compared to 2D cultures, MCTS better recapitulate the characteristics of *in vivo* HNSCC by phenotyping the original tumor regarding cell heterogeneity and genetic variability (61, 62), so that *in vitro* drug responses of the model can be traced back to genetic alterations of the primary tumors (63). Furthermore, MCTS also allowed an enrichment of cancer stem cells as shown for FaDu cells as a model system, thereby offering a suitable tool for active screening for drugs targeting cancer stem cells (64).

**FIGURE 2:**
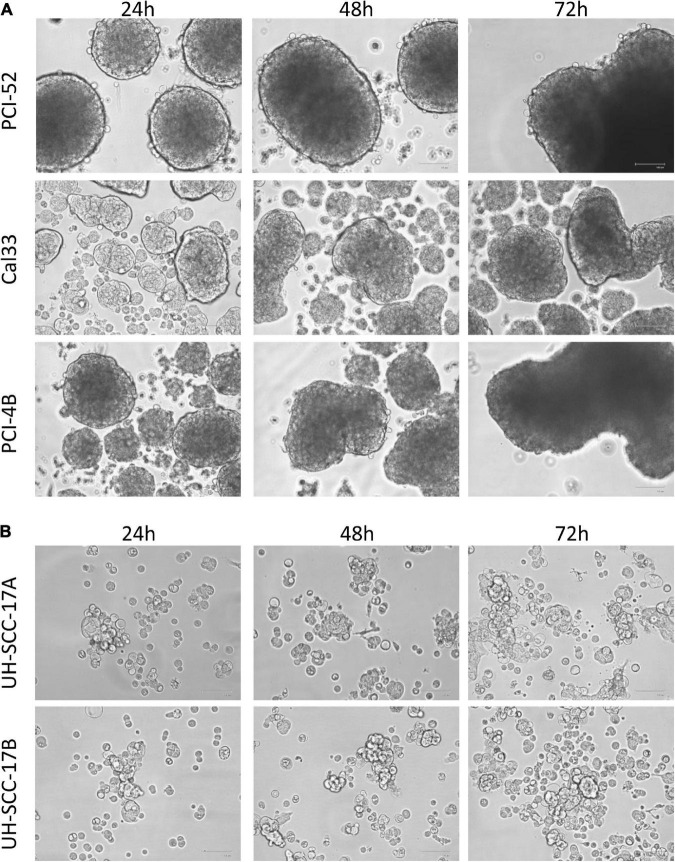
Distinct 3D morphology of head and neck squamous cell carcinoma (HNSCC) cell lines. Representative photos from three different HNSCC cell lines **(A)** and from short-term cultures of a primary and metastatic lesion **(B)** are shown demonstrating the different shape and size of multicellular tumor spheroid (MCTS) obtained from HNSCC cell lines. Magnification: 100×.

There are various methods to proof and to maintain spheroids in culture. First spheroids were generated from single cell suspensions of established 2D HNSCC cell lines by placing them in specific non-adherent, ultra-low attachment plates. The frequency to yield spheroids is dependent on the cell line analyzed. In addition, growth properties, morphology and size differ in the 3D spheroids of HNSCC cell lines (Figure 2). Since they are resembling at least partially the *in vivo* situation, gradients of nutrient and oxygen and physiological cell-to-cell interactions are available. Although 3D MCTS spheroids have advantages compared to 2D monolayer cultures (65), the system has inherent limitations, such as a still simplified architecture due to the lack of TME and crosstalk of immune with tumor cells and reduced long-term maintenance.

Despite these models provided important insights into HNSCC biology and therapy response, they are lacking the potential for a personalized approach, since the stromal cell composition and the diverse TME is missing (66). Recently, two studies have reported on the generation of 3D HNSCC cultures, giving an initial description of this technology, and demonstrating changes regarding cell proliferation, response to anti-cancer therapy as well as expression of genes involved in epithelial mesenchymal transition (EMT) and cancer stem cell features (56, 60). In 2013, it was demonstrated that there is great similarity of EGF-R signaling and radiation response between 3D HSCC cultures and HNSCC xenografts compared to cell monolayers (67). The authors describe a greater physiological relevance of 3D growth conditions regarding the cell morphology, gene and protein expression patterns, protein-protein interactions and intracellular signaling as well as response to radio/-chemotherapy (68, 69). These scaffold-based 3D HNSCC cultures rely on the use of specific ECM, such as Matrigel, Myogel, polyethylene glycol, or synthetic matrices (70, 71), which act as reservoirs of growth factors, but negatively interfere with drug penetrance and alter drug response.

## Organoids of head and neck squamous cell carcinoma

Another option for studying the TME are organoids, which have been established from disaggregated carcinomas from athymic mice for the first time in by Kopf-Maier et al. (72). Over the last decade, different protocols to grow organoids and MCTS from adult human tissues have been established for single-layered (simple) or (pseudo)stratified epithelia from the colon, intestine, liver, pancreas, stomach, esophagus, prostate, lung, breast, fallopian tube, oral epithelium as well as for cancers derived from these tissues (73). In general, organoids develop from stem cells or organ progenitors, and consist of multiple types of organ/tumor-specific cells. Organoids are obtained by embedding fresh dissociated tumor material in suspension or in ECM, such as Matrigel, in the presence of specifically supplemented growth medium. This technology is time consuming, has high costs due to the supplements, and requires well-established protocols for standardization as maintenance is tumor type dependent. A huge advantage of this method is that tumor organoids can phenocopy the *in vivo* tumor characteristics thereby allowing their use for monitoring *in vitro* drug responses linked to genetic alterations present in the original tumor (55, 74, 75), which can be also correlated to the individual clinical outcome and to the response of the corresponding tumor organoids (74, 76). However, it should be noted that next to the complexity of their establishment and manipulation, the size of organoids can largely vary thereby influencing the drug penetrance, efficiency, and reproducibility of results (66, 77). In contrast to spheroids, organoids can be cultured for a long period by preserving its tumor characteristics, which allows their cryopreservation and the development of a patient individualized tumor bank.

Concerning HNSCC, the first organoid cultures without the use of animals were described in and were obtained from surgical resections of patient derived HNSCC samples (78). The tumoroids grow rapidly and in dense structures comparable to that of normal wild-type epithelial organoids or as cystic structures with heterogeneous morphologies between patients and exhibit specific histomorphology changes (79, 80). Furthermore, they have been shown to recapitulate genetic alterations and functionality (61), but it has not yet been analyzed in detail whether the level of cell heterogeneity of HNSCC is kept in the organoids overtime (77, 81). Interestingly, organoids model was shown as a promising tool in predicting the HNSCC patients’ response to radiotherapy (77). Despite the small patients number (*n* = 7), this is a promising start for using organoids model for personalized drug testing.

## Tissue slice (histo) cultures of head and neck squamous cell carcinoma

Tissue slice cultures have been previously described as an appropriate tool for monitoring drug efficacies and the development of personalized treatment strategies. For this, immediately after surgical resection, tumor samples left intact by mechanically cutting or slicing, are then incubated under standardized culture conditions for 24 h, followed by treatment with different therapeutics. Dose response and individual susceptibility can be monitored. Since no tissue processing is performed, the tumor heterogeneity including the stromal and immune cells can be maintained as *in vivo*. This approach has been employed by using tumor slices from human HNSCC xenografts and was used to evaluate their sensitivity to cytotoxic drugs (82). In addition, the radiosensitivity of HPV^+^ HNSCC cells in combination with ATM inhibition was analyzed with this approach and demonstrated an intrinsic double strand break deficiency (83). However, it is noteworthy that this strategy requires relatively voluminous tumor tissue, which is difficult to maintain over 10–14 days and is also not suitable for high throughput analysis (84).

## Microfluidic chip technology in head and neck squamous cell carcinoma

Microfluidic chips have emerged as a promising tool in molecular biology research in general and in the cancer field more specifically. The technology has been most famously used to recreate a human organ-like living environment referred to as “organs-on-chips,” simulating the physiological and pathological features and thus provides a precisely controlled culture environment. Additionally, microfluidic chips have entered several research fields including cancer immunotherapy. While adding stroma cells, such as cancer-associated fibroblasts (CAF) and endothelial cells, to cancer cell cultures already increases the difficulty in setting up culturing conditions, the difficulty increases significantly and becomes challenging when non-adherent immune cells such as CD4^+^ T cells, CD8^+^ T cells, and NK cells are included. This results from differences in the adherence behavior between cancer and immune cells and the difficulty in simulating the movement technique used by the immune cells to migrate from the bloodstream to the tumor site through micro vessels. Given all these challenges, researchers found that microfluidic chips represented a successful approach to studying the crosstalk between cancer and immune cells, as well as a possible assay for testing personalized immunotherapies.

The success of the microfluidic chip as a cell culture assay is linked to several important factors. Microfluidic chips are easy to handle and small. Due to their small size, only a few cells are needed, which is quite important when handling patient’s primary cells, which are difficult to obtain in a high quantity without expanding them in culture. Since the chips are also easy to fabricate, they can be designed based on the specific aim of the assay. This flexibility is unfortunately absent from most *in vitro* and *in vivo* assays. The microfluidic chip provides high-quality imaging and given its thin and tiny structure, different types of microscopes ranging from simple and basic to advanced confocal microscopes can easily be used to image the chips. In addition, microfluidic chips are also suitable for live imaging and can be scanned to yield a full image of the chips. Although microfluidic chips hold all of these advantages and more, they also have drawbacks like any other assay. Among these drawbacks is the difficulty in running high throughput screening in several designs, in addition to the usage of a media flow that is still limited due to the large size of the pumps, restricting the number of chips that can be used in each assay.

Interestingly, several studies reported the use of microfluidic chips to examine different characteristics of cancer-immune cell crosstalk (85–87). For instance, Huang et al. (88) studied the interaction between macrophages and breast cancer cells, while Lucarini et al. (89) designed a chip to study the interaction between immune and melanoma cells and to test the efficacy of type I interferons, a model Parlato et al. (87) also employed to study dendritic cells. A microfluidic chip was also used to study the co-culture of HNSCC cells with two different immune cell types, CD4^+^ T cells and CD8^+^ T cells, through a three-color staining system (90). That same study analyzed the cytokine profile of CD4^+^ T cells, CD8^+^ T cells, and NK cells in the presence of HNSCC cells with or without immune checkpoint inhibitors (PD-1 antibody and IDO-1 inhibitor).

After approving immunotherapy to treat HNSCC, researchers began looking for a biomarker or testing assay to predict patients’ responses to immunotherapy, particularly since HNSCC patients exhibit a low response rate (less than 20%) to such treatments (91). For instance, Al-Samadi et al. (86) introduced the first *in vitro* fully humanized microfluidic chip for the personalized testing of immunotherapies to treat HNSCC. This chip harbors several advantages, beginning with being a fully humanized assay since the chip is supplied with individual patients’ cancer cells, immune cells, and serum together with the human-derived matrix myogel/fibrin. Myogel is a human tumor–derived matrix extracted from leiomyoma tissue (92). Adding the myogel/fibrin matrix increases the reliability of the assay by, first, providing a 3D structure and, second, by rendering the surrounding environment closer to the actual *in vivo* environment of the human tumor. Moreover, Wahbi et al. (93) demonstrated that the HNSCC cells’ behavior and phenotype significantly differ by only changing the culturing matrix. In addition, myogel reportedly increases the reliability of *in vitro* drug testing when compared with patients’ responses (94). Interestingly, these chips could also be used to study a combination therapy, consisting of immunotherapy and chemo-, radio-, and targeted therapies. The chip assay is easy to handle, and the results can be processed within 7 days, making it ideal for integration into patient treatment planning. As with other testing assays, this assay requires validation through a head-to-head comparison with patients’ clinical responses, which is still challenging given the low number of HNSCC patients who receive immunotherapy.

Tumors have a hypoxic microenvironment, especially in the tumor center. Several studies revealed that hypoxia manipulates the behavior of the immune cells toward cancer cells (95, 96). Therefore, it is important to take this element into consideration when studying the crosstalk between cancer and immune cells and the efficacy of immunotherapy. Microfluidic chips offer this feature either through placing the chips in a hypoxia chamber or through passing sodium sulfite as the oxygen scavenger under the chips (97).

**FIGURE 3:**
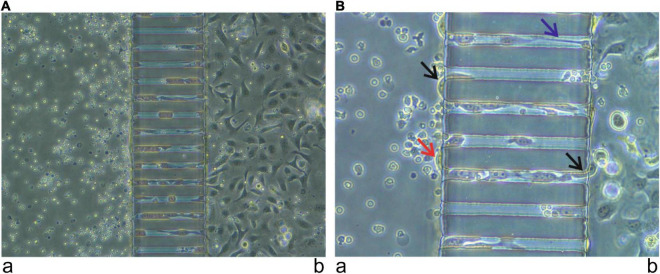
Simulation of metastasis using a microfluidic chip. Immune cells from a healthy donor, provided by the Finnish Red Cross, were injected into the channel (a) and oral tongue squamous cell carcinoma cells (HSC-3) were injected into the channel (b). After 24 h, cancer cells started to pass from channel (b) to channel (a) using the micro-channels. Images were taken using 20× **(A)** and 40× **(B)** magnification. Cells entering and leaving the micro-channels (black arrow). Cells attempting to communicate inside the microchannel (blue arrow). Cells successfully passed from channel (b) to channel (a) (red arrow).

Metastasis is the primary cause of death in cancer since patients rarely die because of primary tumors. HNSCC is characterized by early metastasis, typically diagnosed after metastasis to the regional lymph nodes. Metastasis is a complex process, which begins with cell invasion through the basement membrane structures, followed by intravasation into the vascular or lymphatic system. Finally, some cancer cells extravasate the vasculature to the metastatic sites and are colonized there. The entire process is quite difficult to simulate in most *in vitro* assays. However, the microfluidic chip could mimic this process as cancer cells try to pass through microchannels, areas usually smaller than cancer cells. Thus, the cells change their shape to become cylinders and begin rolling at the wall of the channel in a similar movement to cancer cells in real metastasis (Figure 3). The simulation of the metastasis could even be enhanced by adding endothelial cells to the chip to form a real vessel. One example of this model was created by Pradhan et al. (98), who fabricated a 3D chip-based breast cancer mimetic platform using a tumor-mimetic microvascular network. Their model replicated the pathophysiological architecture of native vascularized breast tumors. In brief, the microfluidic chip technology has opened a new era in the study of immunological characteristics of HNSCC with the potential for further developments in the future.

In sum, the main drawback of using this approach in the culture setup is that all these factors increase the complexity substantially. Furthermore, it is noteworthy that the costs are still high for the setup of the microfluidic system and the read-out methods are still limited.

## Conclusion

Recently, a number of *in vitro* models have been established and could be used for testing novel innovative therapeutic approaches, drug screening, development of individualized therapies, the identification of resistances, novel drug targets, and biomarkers predicting therapy outcome. Organoids and microfluidic systems contributed to an increased understanding of the pathophysiology and the therapeutic effects of drugs on individual patients. While these models seem promising in predicting the patients’ response to anti-cancer treatments, the data supporting them are still very limited and mainly miss the head-to-head comparison with the patients’ response. With more research in this field, these technologies provide novel possibilities to study HNSCC and thereby might provide guidance for patients’ personalized treatment.

## Author contributions

All authors wrote and discussed the manuscript and approved the submitted version.

## Abbreviations

2D: two-dimensional
3D: three-dimensional
CAF: cancer-associated fibroblast
ECM: extracellular matrix
EGF-R: epidermal growth factor receptor
EMT: epithelial mesenchymal transition
HNC: head and neck cancer
HNSCC: head and neck squamous cell carcinoma
HPV: human papilloma virus
iCP: immune checkpoint
iCPI: immune checkpoint inhibitor
IFN: interferon
MCTS: multi-cellular tumor spheroid
OS: overall survival
PD-1: programed cell death-1
PD-L1: programed death ligand 1
TME: tumor microenvironment.
